# Validation of the Substance Use Risk Profile Scale in Lithuanian population

**DOI:** 10.1186/s12955-020-01527-5

**Published:** 2020-08-12

**Authors:** Migle Kaminskaite, Aiste Pranckeviciene, Adomas Bunevicius, Jovita Janaviciute, Darius Jokubonis, Aistė Plioplyte, Inesa Lelyte, Liuda Sinkariova, Ramunas Jokubka

**Affiliations:** 1 Neuroscience Institute, Lithuanian University of Health Sciences, Kaunas, Lithuania; 2 Department of Psychology, Vytautas Magnus University, Kaunas, Lithuania; 3 Republican Centre for Addictive Disorders, Kaunas, Lithuania

**Keywords:** Substance Use Risk Profile Scale, Alcohol dependence, Hazardous alcohol use, Scale adaptation, Personality

## Abstract

**Background:**

Personality traits are related with risk of hazardous alcohol use and alcohol dependence. The Substance Use Risk Profile Scale (SURPS) measures personality traits associated with addictive substance abuse. We examined psychometric properties of the SURPS in Lithuanian population.

**Materials and methods:**

Two hundred forty-seven participants (mean age 37.22 ± 0.78 years), were recruited from the local community and from an inpatient addiction treatment centre. Internal consistency, stability, factor structure, content validity, and external validity of the SURPS were examined. Hazardous alcohol use was evaluated by Alcohol Use Disorder Identification Test (AUDIT). Alcohol dependence diagnosis was established by International Classification of Diseases - 10 (ICD - 10). We also performed gender analyses for associations of personality traits with alcohol dependence and hazardous use of alcohol.

**Results:**

The SURPS scale demonstrated appropriate internal validity, good temporal stability, and adequate criterion validity and construct validity. The SURPS scores of hopelessness, anxiety sensitivity and impulsivity were higher in the alcohol dependence group than in the control group for both males and females. Impulsivity and sensation seeking were associated with hazardous alcohol use and these associations were more prevalent in females.

**Conclusions:**

Lithuanian translation of the SURPS scale was appropriate. The SURPS demonstrated good sensitivity for discriminating on alcohol dependence and was more sensitive for discriminating on hazardous alcohol use for females.

## Introduction

Substance use disorders manifest as compulsive seeking and consumption of addictive substances despite harmful consequences [1]. The Diagnostic and Statistical Manual of Mental Disorders – 5 (DSM-5) describes substance use disorder as hazardous use of addictive substances causing social and interpersonal problems and neglect of major social roles [1]. Substance use induces tolerance, loss of consumption control, unpleasant withdrawal and reduced physical and mental health [1]. It is established that several personality traits are related with hazardous addictive substance use and subsequent dependence development [2, 3].

High rates of alcohol consumption is an important public health problem in Lithuania as the country has one of the highest alcohol consumption rates in Europe [4]. Better understanding of diverse pathogenesis of addiction disorders and predisposing factors would be beneficial in the development of more advanced prevention and treatment methods of addiction disorders [5]. Several questionnaires for evaluating personality traits and divergent psychological phenotypes associated with addiction risk were created, for example the Minnesota Multiphasic Personality Inventory [6], Tridimensional Personality Questionnaire [7] and Addiction Prone Personality Scale [8]. However, there is a shortage of specific substance abuse related psychometric instruments validated in Lithuanian population.

The Substance Use Risk Profile Scale (SURPS) was developed to reveal main reinforcement mechanisms of addictive substances use [9]. Addiction is multi-ethiological cluster of syndromes, therefore divergent phenotypes are expected. Different personality traits that predispose to vulnearability may also affect motivational cues of addictive substance use, choice of addictive substance, and risk for relapse [5, 10–12]. Better understanding in diverse pathogenesis of addiction disorders and predisposing factors would be beneficial in the development of more advanced prevention and treatment methods of addiction disorders [5].

Generally, two main theoretical motivation dimensions are distinguished: a positive reinforcement, that could be defined as higher reward dependence that manifests in approach behaviour; and a negative reinforcement, which reveals as seeking escape and avoidance of aversive stimulus [11, 13]. Addictive substances per se have rewarding properties [14], thus higher reward dependence may result in increased vulnerability to addictive disorders. Poor behavioural inhibition and self-regulation capacities are classical findings in studies of addiction prone personality [2, 3]. Moreover, the presence of attention deficit – hyperactivity disorder (ADHD) has been demonstrated to increase vulnearability to addiction development [15, 16]. These findings are anticipated, as addiction disorders manifest in loss of control upon use and compulsive consumption pattern. Impulsiveness is predisposed by neurobiological alterations and enhanced by the abuse of addictive substances [17]*.* In addition, sensation seeking can lead to experimentation with addictive substances that consequantially opens gates for addiction [18].

Another pathway to addiction might be related to negative affect, and hopelessness, depression and anxiety may also escalate the use of addictive substances [19, 20] especially when trying to cope with negative emotional states. Comorbidity of substance abuse and mental disorders with predominant negative affect is well known [21, 22] and chronic use of addictive substances can increase the susceptibility for negative affect by activating the hypothalamic-pituitary-adrenal axis [23].

The SURPS covers these two theoretical motivational dimensions of addiction by evaluating personality traits associated with the substance abuse and addiction risk, in particular: anxiety sensitivity, hopelessness, impulsivity and sensation seeking. Respondents scoring high on anxiety sensitivity and hopelessness subscales of the SURPS are more likely to use addictive substances in order to eliminate negative emotions, while respondents receiving high impulsivity and sensation seeking scores tend to use addictive substances in order to enhance positive emotions [9]. Thus, the SURPS questionnaire is useful for studying mechanisms of addictive substance consumption. Moreover, the scale includes 23 questions, is self-administered, brief and easy to apply which is beneficial in research studies.

The scale was validated in adolescents and young adults populations and has shown good reliability and also a predictive value in longitudinal studies [24]. The scale has demonstrated high intercultural validity and reliability, and is adapted for different languages, including French [24–26], English [9, 24, 26], Irish [24], German [24], Dutch [27], Bulgarian [28], Spainish [29, 30], Turkish [31], and others. However, only few studies included patients with clinical diagnosis of addiction [28, 32, 33].

The aim of our study was to validate the SURPS questionnaire in the Lithuanian population and to explore the psychometric properties of the scale. We investigated personality traits associated with hazardous alcohol use and dependence and evaluated the properties of the scale in middle-aged adults. Different patterns in hazardous alcohol use in males and females [34] encouraged us to examine divergent traits associated with addiction and hazardous use of alcohol between genders.

## Methods

### Participants

The study sample included 247 persons: 178 healthy volunteers, recruited from the local community by convenience and snowball sampling methods, and 69 patients diagnosed with alcohol dependence who were recruited from the Republican Centre for Addictive Disorders.

Study inclusion criteria were willingness to participate, age from 20 to 65 years, ability to provide written informed consent and comprehend Lithuanian. The study exclusion criteria were pregnancy and severe cognitive impairment that would interfere with ability to understand the study aims and procedures. The patients in treatment were included if they had alcohol use disorder according to the International Classification of Diseases - 10 (ICD-10) diagnostic criteria (F10.2, F10.3). Diagnosis of alcohol use disorder was established with respect to diagnostic criteria defined in ICD-10 by a psychiatrist who specialises in addiction disorders. Patients with polysubstance abuse (*N* = 8) were included in the study.

### Procedure

The study protocol and consent procedures were approved by the Regional Bioethics Committee for Biomedical Research (Nr. BE-2-25). All participants signed written informed consent with ability to discontinue participation at any time. Participants were approached twice during the study. During the first visit respondents were asked to provide demographic data and to complete a battery of study questionnaires. Respondents were also asked to participate in a re-test survey of the SURPS scale and provide their contact details. Twenty-six participants were re-evaluated at 1 month after inclusion in the study. At both time points questionnaires were administered by paper-pencil method.

### Instruments

#### Substance Use Risk Profile Scale

The SURPS scale contains 23 items measuring four personality traits related to substance abuse: hopelessness (7 items), anxiety sensitivity (5 items), impulsivity (5 items) and sensation seeking (6 items) [9]. It is a self-report questionnaire with possible answers to all questions ranging from 1 (strongly disagree) to 4 (strongly agree). All questions are scored by adding scores together with the exception of the hopelessness subscale which items are scored reversed. Higher score on each subscale indicates greater expression of the personality traits.

#### Barratt impulsiveness Scale – 11

The Barratt Impulsiveness Scale – 11 (BIS − 11) is a 30 – item self – report questionnaire designed to measure impulsivity with its three main factors: lack of attention, motor impulsivity and non-planning [35]. Participants respond on a 4 – point Likert-type scale with possible scores ranging from 1 (Rarely/Never) to 4 (Almost always/Always). Higher score indicates a greater level of impulsivity [35]. According to previous studies, internal consistency of this scale is acceptable (Cronbach alpha = 0.79) [36]. In our study the internal consistency of BIS-11 scale by Cronbach’s coefficient alpha was 0.82. The permission to use this scale in current research was obtained from scale copyright holder. The BIS-11 was adapted for use in Lithuania using a standard double-translation method by the study researchers [37]. The BIS-11 scale was used to evaluate validity of the SURPS subscale of impulsivity.

#### Ten item personality measure

The Ten Item Personality Measure (TIPI) consists of 10 items that measure the Big-Five personality domains: extroversion, agreeableness, conscientiousness, emotional stability and openness to experiences. Two items are used to evaluate each trait. Respondents are asked to specify how strongly he/she agrees with the given statement. Each item is rated on a 7 – point scale ranging from 1 (disagree strongly) to 7 (agree strongly). Lithuanian version of the TIPI has previously demonstrated good construct validity [38, 39].

The conscientiousness factor was chosen to evaluate validity of the SURPS subscale of impulsivity, emotional stability – the subscales of hopelessness and anxiety sensitivity, openness to experience factor - the subscale of sensation seeking, as these traits considerably overlap [40].

#### Patient Health questionnaire - 4

The Patient Health Questionnaire - 4 (PHQ-4) [41] is a 4 item inventory designed for brief assessment of depressive and anxiety symptoms. The first two items of the PHQ – 4 measures anxiety symptoms and the last two items measure the depressive symptoms. Responses are scored from 0 to 3 (0 = Not at all; 1 = Several days; 2 = More than half the days; 3 = Nearly every day).. Internal consistency by the Cronbach’s coefficient alpha was more than 0.8 in line to previous studies [41]. Internal reliability of the PHQ-4 in our study was 0.90. The PHQ-4 and its subscales are adapted and used in Lithuania [42]. In this study the PHQ-4 was used to evaluate convergent validity of the SURPS hopelessness and anxiety sensitivity subscales.

#### Alcohol Use disorder identification test

Hazardous alcohol consumption was evaluated using the Alcohol Use Disorder Identification Test (AUDIT) [43]. This 10-item scale measures three conceptual domains: frequency and amount of alcohol intake (items 1–3), dependence (items 4–6) and adverse consequences (items 7–10). Scale scores range from 0 to 40 points. Cut-off point of the scale is 8, which indicates a potential problem of alcohol use and risk of alcohol use disorder [43]. The AUDIT was developed by the World Health Organization (WHO) and is a freely accessible, valid, and reliable instrument which demonstrates high sensitivity and specificity for hazardous alcohol use [44]. The AUDIT was used to evaluate the external validity of the SURPS in our study.

#### Translation and adaptation procedure of the SURPS

Translation and adaptation procedure of the SURPS scale was implemented according to the second edition of the International Test Commission for translating and adapting tests [37] after obtaining a written permission from the copyright holder.

Translation of the SURPS to Lithuanian language was done by a native Lithuanian speaker medical doctor specializing in substance use disorders. A pilot study in 20 respondents was performed to assess the appropriateness and clarity of the translation. Minor corrections in question wording were made after the review of this pilot study results in research group discussions. Two independent translators made back-translations to the English language to determine any discrepancies. Translators were fluent in Lithuanian and English and were blinded to the original scale. After subsequent review of the translations and when consensus on translation accuracy was reached, Lithuanian translation of the SURPS was submitted to the author of the SURPS scale.

#### Statistical analysis

Statistical analysis was performed using the SPSS statistical package (IBM SPSS Statistics 20, Chicago, IL) and confirmatory factor analysis was performed using the AMOS statistical package. Nonparametric criteria were applied where appropriate because of not normal distribution of the SURPS subscale scores.

Internal consistency of the total SURPS scale and individual subscales was estimated by the Cronbach’s coefficient alpha, with value of 0.7 considered as acceptable [45]. Spearman’s correlation coefficients of the SURPS subscales with corresponding scales were examined to determine content validity.

Test-retest reliability of the SURPS was evaluated by the Spearman correlations for individual subscales and by mixed model intra-class correlation coefficient (ICC) for absolute agreement. ICC values below 0.5 were considered as poor, 0.5–0.75 as moderate, 0.75–0.9 as good, and > 0.9 as excellent [46].

Maximum likehood estimation confirmatory factor analysis (CFA) [47] with bootstrapping of 250 iterations was conducted to verify four factor structure of the SURPS. Model goodness-of-fit was estimated by relative χ^2^, Comparative Fit Index (CFI), Root Mean Square of Approximation (RMSEA), Normed Fit Index (NFI). Relative χ^2^ < 2, CFI > 0.9 and RMSEA < 0.08, NFI > 0.8 were considered as acceptable [48]. Covariances between factors were included in the model, because personality traits correlated in original scale and in scales validated in other populations. Significant covariances of items belonging to the same factor that resulted in better model fitting were included to model [9, 30]. Items with low weigh loading (< 0.4) were removed from the model. Configural invariance analysis stratified by gender was performed by testing the same model in males and females and by performing a combined group analysis [49]. Metric invariance was evaluated by constricting factor loadings for groups stratified on gender [49].

Clinical diagnosis of alcohol use disorder according to the ICD-10 criteria and the AUDIT results were used to establish discriminative validity of the SURPS. Participants were divided into three groups according to clinical diagnosis of alcohol use disorder and the AUDIT score: control group (AUDIT score < 8), hazardous use of alcohol (AUDIT score > 8 and no clinical diagnosis of alcohol use disorder) and alcohol dependence group (clinical diagnosis of alcohol use disorder). Differences between groups were evaluated by Kruskal – Wallis test. Post-hoc analysis for pairwise comparisons was performed with *p*-value adjustment for multiple comparisons.

Relative operating characteristic curves (ROC) were applied to evaluate sensitivity and specificity of the SURPS scale for alcohol dependence and hazardous alcohol use [50].

## Results

### Participants

The final sample consisted of 247 participants, as 4 of volunteers and 3 patients were excluded due to insufficient data (e.g. have not filled some of the scales) and included 108 males and 139 females. Mean age of participants was 37.22 (± 0.78) years. Proportion of females was higher in the control group and proportion of males was higher in the alcohol dependence group. Demographic and clinical data of participants are presented in Table 1.

**Table 1:** Demographic and clinical data of participants

	Control group	Particpants with hazardous use of alcohol	Patients with alcohol dependence	Statistics	***P*** value
				χ^2^,df	
**Total sample**	140	38	69		
Females, (%)	101 (72.1%)	18 (47.4%)	20 (29.0%)	36.43 (df = 2)	< 0.001
Males, (%)	39 (27.9%)	20 (52.6%)	49 (71.0%)		
				F, (df)	
**Age, mean, (SD)**	35.64 (12.54)	34.00 (12.07)	42.22 (10.12)	8.81 (df = 2)	< 0.001
				χ^2^,df	
**Education**					
Basic/secondary, (%)	34 (24.3%)	10 (26.3%)	53 (76.8%)	56.63 (df = 2)	< 0.001
High school/ University, (%)	106 (75.7%)	28 (73.7%)	16 (23.2%)		
**Marital status**					
Single, (%)	33 (23.6%)	10 (26.3%)	23 (33.3%)	2.25(df = 2)	0.32
In relationship, (%)	107 (76.4%)	28 (73.7%)	46 (66.7%)		

*SD* standard deviation; Hazardous use of alcohol – persons at risk of alcohol use disorder by cut-off value of 8 of Alcohol Use Disorder Identification test, but without established alcohol dependence diagnosis; *df* degrees of freedom

### Psychometric properties of Substance Use Risk Profile Scale

#### Structural validity

**Fig. 1:**
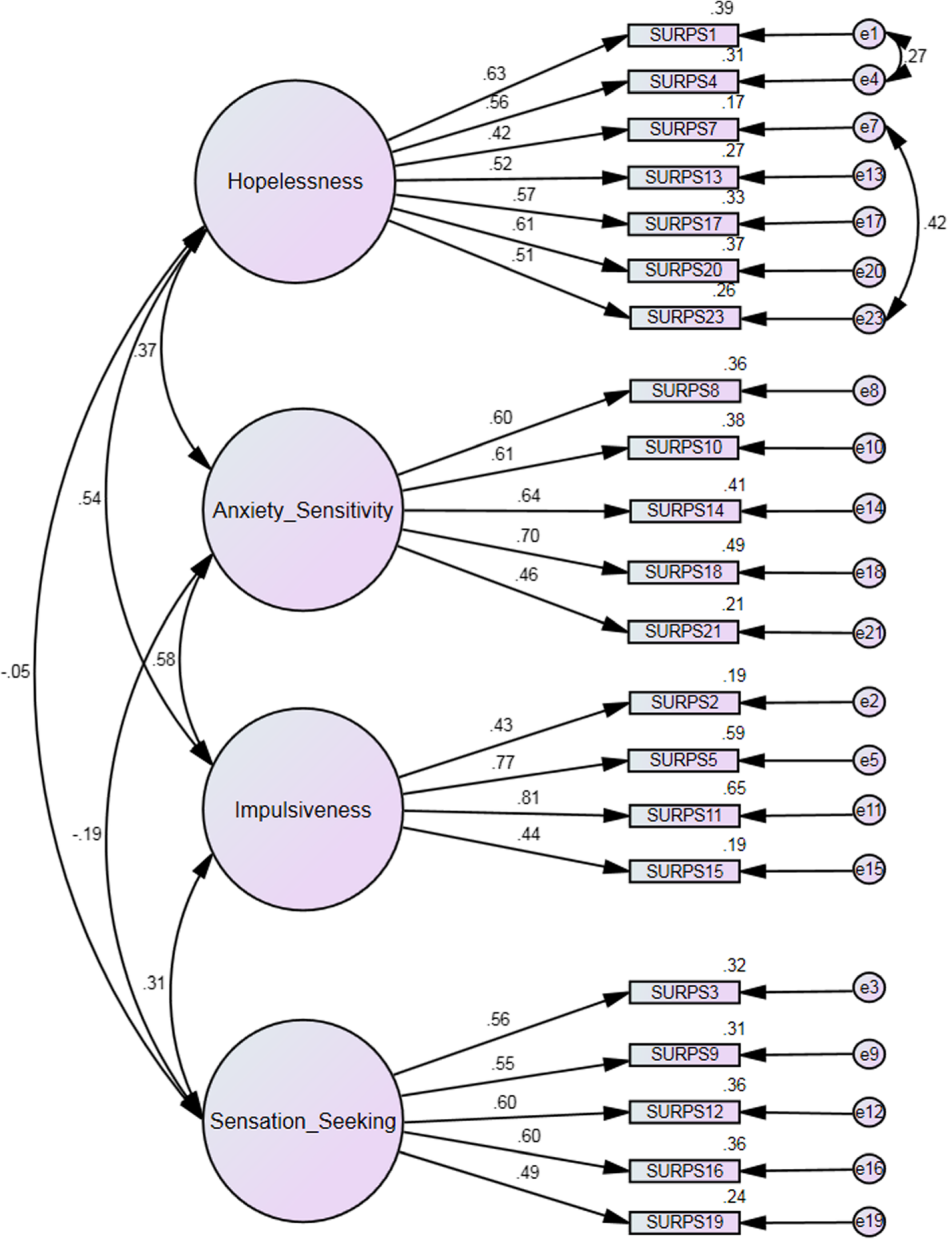
Four factor structure of the Substance Use Risk Profile Scale with correlations of factors

CFA without included covariances of items resulted in a poor model fit: χ^2^ (df = 224) = 462.80, *p* < 0.001, relative χ^2^ = 2.06, CFI = 0.817, RMSEA = 0.066, NFI = 0.703). Items 22 and 6 had sub-threshold loading weights (< 0.4). Removal of items 6 and 22 and specifying covariances that were suggested by modification indices (between items 1 and 4, and 7 and 23), resulted in improved model fit: χ^2^ (df = 181) = 353.37, *p* < 0.001, relative χ^2^ = 1.95, CFI = 0.863, RMSEA = 0.062, NFI = 0.759). Even though the model fit indices CFI and NFI were slightly below desirable values, we relied on relative χ^2^ and RMSAE, which are less sensitive to sample size [51], which indicated an appropriate model fit. Four factor structure of the SURPS with included covariances of items is presented in Fig. 1.

Examination of factor covariances showed significant correlations between impulsivity and hopelessness, impulsivity and sensation seeking, impulsivity and anxiety sensitivity, hopelessness and anxiety sensitivity, sensation seeking and impulsivity (*p* < 0.05) and significant negative correlation between sensation seeking and anxiety sensitivity. Other tested covariances were insignificant.

Examination of model for groups stratified by gender demonstrated high configural gender non-invariance (Table 2). The model fit was worse in females than males. However, our study sample was limited, the design of study was explorative and RMSEA and relative χ2 were appropriate for both male and female groups. Therefore, we decided not to remove any more questions and performed analyses for males and females based on the same scale structure.

**Table 2:** Configural and metric invariance of the model

Model	χ2	df	CFI	RMSEA	ΔCFI	ΔRMSEA	NFI	Relative χ2
Baseline model	353.37	181	0.86	0.06			0.76	1.95
Males	235.02	181	0.89	0.05	0.03	−0.01	0.67	1.30
Females	350.08	181	0.79	0.08	−0.07	0.02	0.65	1.93
Combined baseline model for males and females	581.06	362	0.83	0.05	0.03	−0.01	0.66	1.62
Combined baseline model for males and females, factor loadings constrained to be equal	606.24	379	0.82	0.05	0.04	−0.01	0.65	1.60

Baseline model – items 6, 22 removed, with covariances 1–4, 7–23 specified

*CFI* Comparative Fit Index, *RMSEA* Root Mean Square of Approximation, *NFI* Normed Fit Index

#### Internal consistency

Internal consistency of the SURPS subscales measured by the Cronbach coefficient alpha ranged from acceptable to good. The Cronbach’s coefficient alpha was 0.78 for hopelessness subscale, 0.71 for anxiety sensitivity subscale, 0.71 for impulsivity subscale and 0.70 for sensation seeking subscale. Cronbach’s coefficient alpha for the total SURPS scale was 0.74.

#### Temporal stability

Spearman correlations and ICCs indicated appropriate test-retest stability of the SURPS. There were strong correlations between the two assessment time points for score of the total SURPS scale (*r* = 0.64, *p* < 0.001), and scores on the SURPS subscales of hopelessness (*r* = 0.65, *p* < 0.001), anxiety sensitivity (*r* = 0.85, *p* < 0.001), impulsivity (*r* = 0.801, *p* < 0.001), and sensation seeking (*r* = 0.75, *p* < 0.001). The ICCs of two assessments documented moderate to good reliability: hopelessness 0.60 (95% CI 0.29–0.80), anxiety sensitivity: 0.80 (95% CI 0.61–0.90), impulsiveness: 0.81 (95% CI 046–0.91), sensation seeking: 0.75 (95% CI 0.52–0.88), total scale: 0. 72 (95% CI - 0.47 – 0.87).

#### Criterion validity

All subscales had medium to strong significant correlation with the corresponding constructs measured using different self-rating instruments. Spearman correlations of applied instruments are presented in Table 3. As expected, we found positive correlations between similar personality traits and negative correlations between opposite traits measured by the SURPS and corresponding instruments.

**Table 3:** Correlations of the scales presenting the construct validity of the Substance Use Profile Scale

	SURPS Hopelessness	SURPS Anxiety Sensitivity	SURPS Impulsivity	SURPS Sensation Seeking
BIS-11 Impulsivity	0.40 **	0.25 **	0.62 **	0.10
TIPI Extroversion	−0.22**	− 0.18 *	0.05	0.13*
TIPI Agreeableness	0.04	0.05	−0.03	− 0.10
TIPI Conscientiousness	−0.25**	**− 0.004**	− 0.30**	−0.08
TIPI Emotional Stability	−0.29**	−0.31**	− 0.38**	0.10
TIPI Openness to Experiences	−0.32**	−0.14*	− 0.02	0.23**
PHQ-4 Depression	0.42**	0.34**	0.42**	−0.05
PHQ-4 Anxiety	0.39**	0.36**	0.44**	−0.04

*SURPS* Substance Use Profile Scale, *BIS – 11* Barratt Impulsiveness Scale 11, *TIPI* Ten Item Personality Measure

*- *p* < 0.05; **- *p* < 0.001

SURPS hopelessness subscale score correlated positively with the PHQ – 4 depression subscale score and negatively with the TIPI emotional stability subscale score; The SURPS subscale of anxiety sensitivity score correlated positively with the PHQ – 4 anxiety subscale score and negatively with the TIPI emotional stability score; the SURPS impulsivity subscale score correlated positively with the BIS-11 score and negatively correlated with the TIPI conscientiousness subscale score; the SURPS sensation seeking subscale score correlated positively with the TIPI openness to experience subscale score. In addition, there were medium positive correlations between internalising factors, as the SURPS hopelessness score correlated with the PHQ-4 anxiety score, and the SUPRS anxiety sensitivity correlated with the PHQ-4 depression subscale score. SURPS hopelessness and anxiety sensitivity scores demonstrated medium positive correlations with impulsivity measured by the BIS-11 and a modest negative correlation with conscientiousness subscale of the TIPI. The SURPS impulsivity subscale had medium positive correlations with the PHQ-4 depression and anxiety subscales and a medium negative correlation with the TIPI emotional stability subscale. Internalising traits of the SURPS negatively correlated with the TIPI experience seeking subscale. The SURPS sensation seeking subscale had a weak correlation with the TIPI extroversion subscale.

#### Sensitivity

Median scores and interquartile ranges of the scores on the SURPS subscales and the total scale and differences between groups of control, hazardous alcohol use and alcohol dependence are presented in Table 4.

**Table 4:** Differences of SURPS scores in participants with alcohol dependence, hazardous use of alcohol and controls

	Control group		Hazardous use of alcohol		Alcohol dependence		
	Mean	Median, [Q1-Q3]	Mean	Median, [Q1-Q3]	Mean	Median, [Q1-Q3]	H, (df = 2)
**Hopelessness**							
Total sample	12.33	12 [10–14]	13.18	12.5 [10–14]	14.83	15** [13–18]	25.32
Males	12.26	12 [10–14]	12.45	12 [10–14]	15.18	15** [13–18]	17.19
Females	12.37	12 [10–14]	13.94	13 [11.75–14.25]	13.95	14 [12.25–16.75]	5.63
**Anxiety sensitivity**							
Total sample	11.86	12 [10–14]	12.76	13 [11–15]	13.32	14** [11–16]	18.03
Males	10.67	10 [9–12]	11.75	12 [9–14]	13.33	14** [11–16]	15.24
Females	12.37	13 [10–15]	13.88	14 [11–15.25]	15.25	15** [13.25–17.75]	14.92
**Impulsivity**							
Total sample	8.33	8 [7–10]	9.00	9 [7–10.25]	10.74	13** [11–14]	41.15
Males	7.85	8 [7–9]	8.35	8 [6.25–10]	10.59	11** [9–12]	25.11
Females	8.51	8 [7–10]	9.72	9.5 [7.75–12]	11.10	10.5** [10–13]	19.65
**Sensation seeking**							
Total sample	12.18	12 [9–16]	13.76	14** [12–16.25]	13.45	13* [11–16]	9.50
Males	13.54	13 [11–16]	13.60	14 [12–16.75]	13.96	14 [12–16]	0.13
Females	11.65	12 [9–14]	13.94	14** [12–16.25]	12.20	11 [8.5–14.75]	6.45
**Total scale**							
Total sample	44.71	44 [41–49]	48.68	48** [43.75–53]	52.90	53** [47.5–58]	55.71
Males	44.31	44 [40–49]	46.15	44.5 [41.25–50.5]	53.06	53** [47–58.5]	31.40
Females	44.86	45 [41–49]	51.50	51** [47.75–54.25]	52.50	52.5** [48–57.5]	26.30

*Q1* quartile 1, *Q3* quartile 3, *df* degrees of freedom

* - *p* < 0.05, compared to control group; **- adjusted *p* < 0.05, compared to control group

Patients with alcohol dependence scored significantly higher than the control group on all SURPS subscales. However, the significance of sensation seeking was not maintained after *p*-value adjustment, and also in gender separate analysis. Stratification by gender revealed that scores of hopelessness were higher in alcohol dependent males but not females.

Hazardous alcohol use as indicated by the AUDIT test cut-off value of 8 was significantly associated with sensation seeking. Stratification by gender revealed that sensation seeking was driven by female group and an association of impulsivity to hazardous use of alcohol was present in females but not males.

**Fig. 2:**
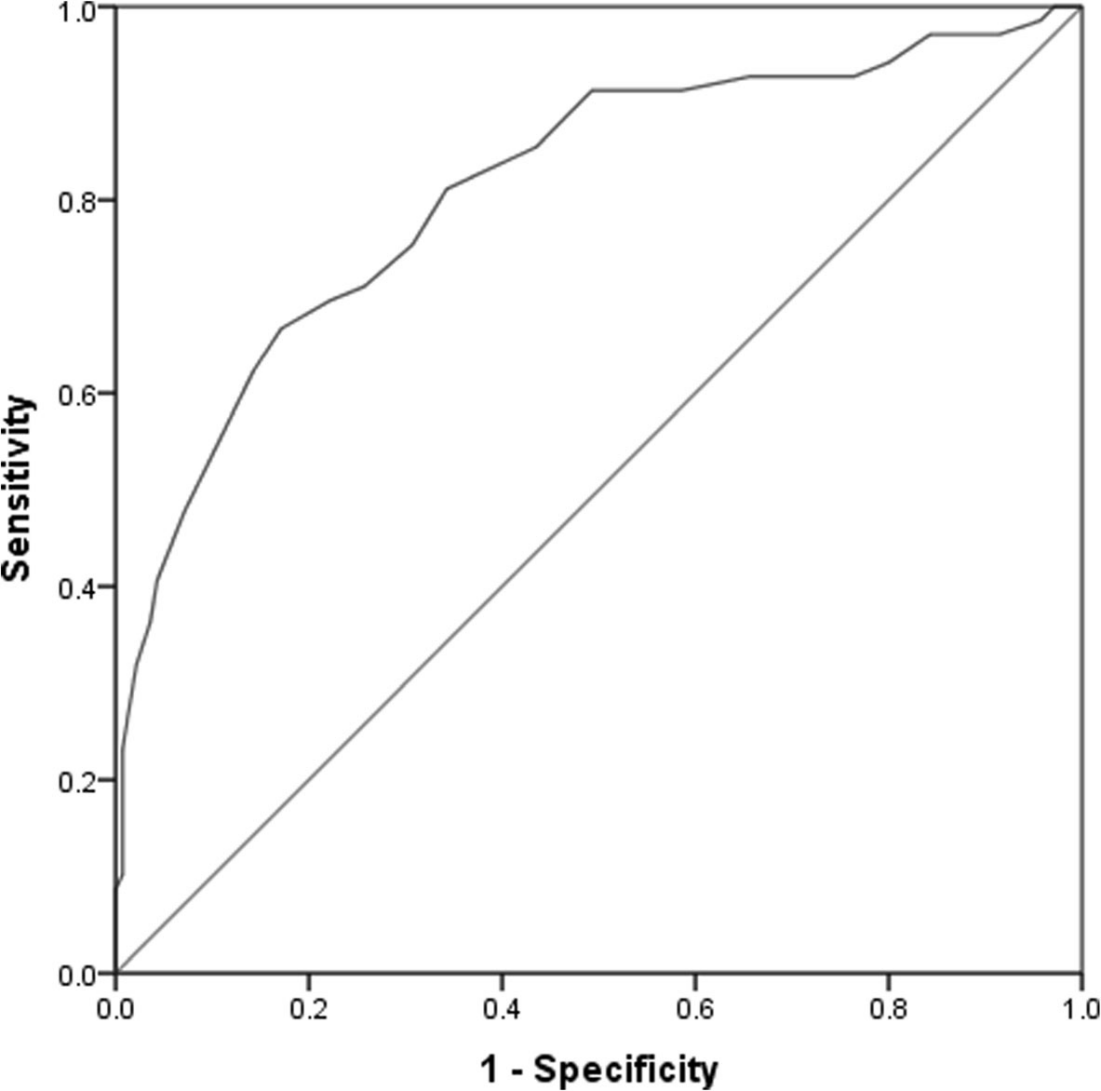
Receiver operating characteristic curve (ROC) for alcohol dependence. Sensitivity of Substance Use Risk Profile Scale measured by Area Under the Curve (AUC) on predicting alcohol dependence. AUC = 0.81, *p* < 0.001

**Fig. 3:**
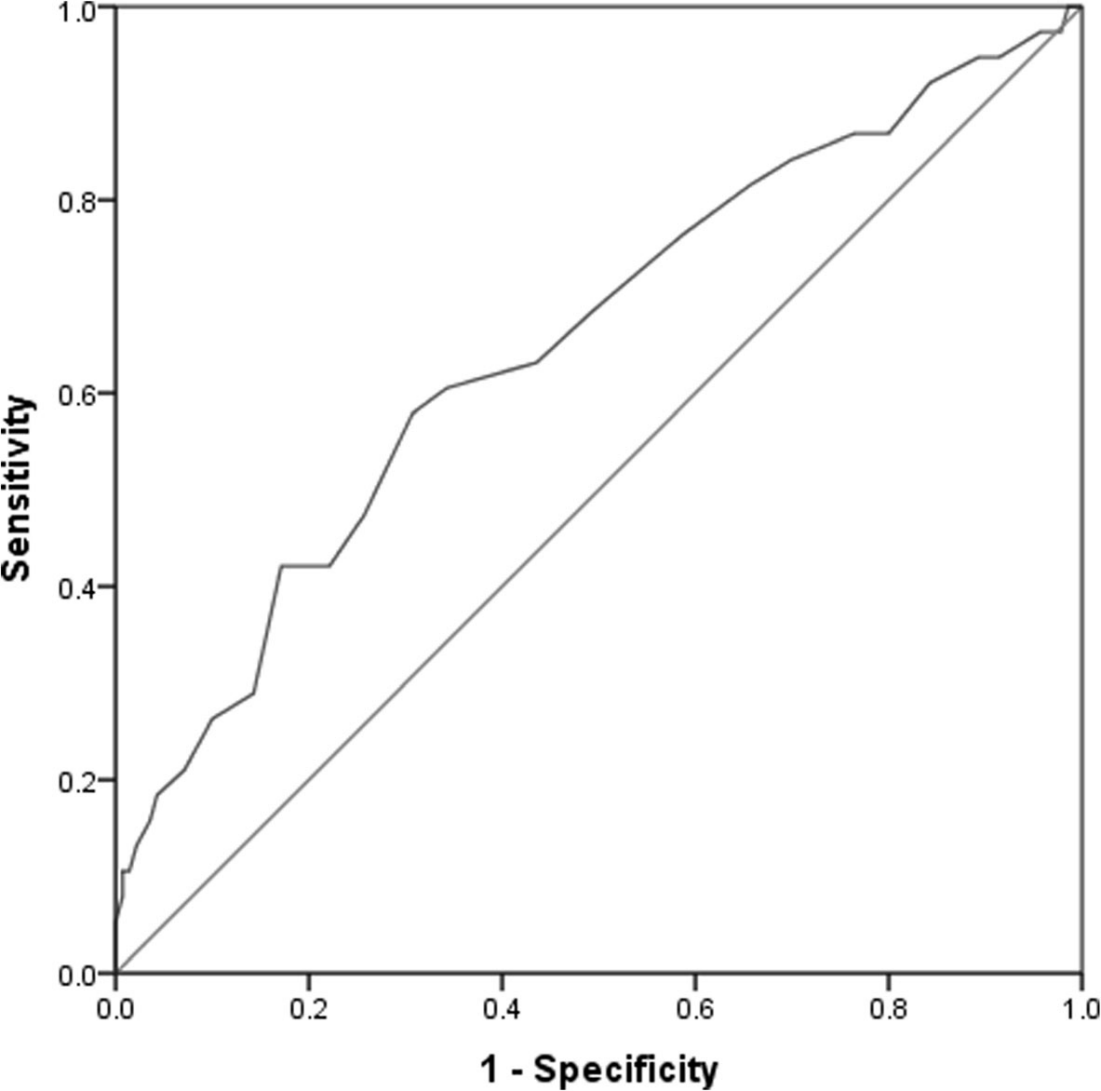
Receiver operating characteristic curve (ROC) for hazardous alcohol use. Sensitivity of Substance Use Risk Profile Scale measured by Area Under the Curve (AUC) on predicting hazardous alcohol use (AUDIT score ≥ 8, but no established diagnosis of alcohol dependence). AUC = 0.65, *p* < 0.05

Accuracy of the SURPS subscales for discriminating groups of alcohol dependence versus control and of hazardous alcohol use versus control measured by areas under the curves (AUC) are presented in Table 5. Investigation of the ROC curves suggested that the SURPS scale had good sensitivity for alcohol dependence (AUC = 0.81; Fig. 2). There were no meaningful differences in the AUCs for alcohol dependant males and females. However, for hazardous alcohol use, the AUC indicated poor sensitivity of the SURPS scale (AUC = 0.65; Fig. 3). Gender analysis suggested that the SURPS scale was more sensitive for females (AUC = 0.78) than for males for predicting hazardous alcohol use (AUC = 0.56).

**Table 5:** Sensitivity of SURPS scale for alcohol dependence and hazardous alcohol use

	Area under the curve (AUC), [95% Cl.]	
	Hazardous alcohol use versus control	Alcohol dependence versus control
Total sample	0.65* [0.55–0.77]	0.81** [0.75–0.88]
Males	**0.56 [0.40–0.72]**	0.83** [0.75–0.92]
Females	0.78** [0.66–0.89]	0.79** [0.67–0.91]

Hazardous alcohol use – participants who scored 8 or more in the AUDIT test but had no established diagnosis of alcohol dependence. Control – participants without alcohol dependence diagnosis and scored less than 8 in the AUDIT

*- *p* < 0.05, for the null hypothesis AUC =0.5; **- *p* < 0.001, for the null hypothesis AUC =0.5

## Discussion

In this study we aimed to evaluate psychometric properties of the SURPS scale in Lithuanian population. The scale demonstrated appropriate internal validity, good temporal stability, adequate criterion validity, and sensitivity. The construct validity of the scale was appropriate after removal of two items. We examined differences of scores of the SURPS subscales in groups stratified by alcohol dependence, hazardous alcohol use and control to evaluate the sensitivity of the scale. Alcohol dependence group scored higher than the control group in hopelessness, anxiety and impulsivity. Association of the SURPS scores with hazardous use of alcohol was marginal and driven by females.

CFA without added correlations between items 1 and 4, 7 and 23, indicated poor model fitting, thus these correlations were added into the model similarly to other studies of the SURPS scale because of similar wording of questions [9, 30]. Item 6 (**“**I enjoy new and exciting experiences even if they are unconventional.”) and item 22 (“I feel I have to be manipulative to get what I want.”) had sub-threshold standardized loading coefficients in the CFA and were excluded resulting in appropriate model fit of SURPS scale according to the RMSEA and relative χ^2^.

Obstacles with the originally proposes four factor structure of SURPS were encountered in prior studies. Specifically, cross-loading of item 16 was also reported in other adaptations of the SURPS scale [26, 31, 33, 45, 52]. Because of cross-loadings, item 16 was removed in a few studies on the SURPS [26, 33, 45, 52], but retained in the Turkish adaptation in order to preserve the original structure of the scale [31]. Moreover, there were suggestions to shorten the scale by removing problematic items: 20 item scale was used in Canada [52] and a 15 item SURPS variant in the USA [53].

The SURPS had appropriate internal consistency and criterion validity. These findings were similar to the original [9] and other SURPS scale validation studies [26, 28–31, 33]. There were small to medium correlations of SURPS internalizing trait subscales (hopelessness and anxiety sensitivity) with impulsivity measured by the BIS-11 and the SURPS impulsiveness subscale. Scores of the SURPS impulsivity subscale had medium correlations with the PHQ-4 depression and anxiety scores. Similar correlation of internalizing traits with impulsivity was also found in other SURPS studies [9, 24, 25, 28, 45]. The tendency for higher hopelessness and anxiety could be associated with higher impulsivity level as impulsive behaviour might reveal itself in negative affects [54]. Diagnosis of alcohol dependence was associated with higher scores on all SURPS subscales except sensation seeking suggesting good sensitivity of the SURPS scale in alcohol dependent individuals. Only a few studies examined properties of the SURPS scale in clinical samples. In Bulgaria, patients were diagnosed with alcohol abuse and dependence according to the DSM-IV criteria [28]. Authors demonstrated associations of impulsivity and sensation seeking with alcohol abuse and an association between impulsivity and alcohol dependence. There was no association of hopelessness and anxiety sensitivity neither with alcohol abuse, nor with dependence in Bulgaria [28]. Nevertheless, in a clinical sample in the USA, hopelessness and anxiety sensitivity were associated with the number of drinks per occasion and alcohol related problems [33]. Moreover, in the USA the internalizing traits were related with both negative reinforcement (coping with emotions) and positive reinforcement (enhancement) motives for consuming alcohol [33]. Whilst the original scale validation study by Woicik et al. [9] have not included clinical samples, authors measured alcohol abuse and dependence symptoms by the Comprehensive Drinker Profile. Alcohol abuse and dependence symptoms were associated with hopelessness, impulsivity and sensation seeking [9]. These divergent findings of personality trait relation to alcohol abuse and dependence suggests inter-cultural differences, and subsequent possibility of the need for different approaches in treatment methods in different populations.

Only a few studies have demonstrated associations of anxiety sensitivity with alcohol use and abuse [9, 51]. The authors of the scale hypothesised that the associations of anxiety sensitivity with abuse of psychotropic substances is more explicit in later stages of addiction development [9]. Our findings agree with this hypothesis, as the scores of anxiety sensitivity subscale were significantly higher in the alcohol dependence group and showed only marginal associations with risky use of alcohol. Chronic alcohol use is known to attenuate hypothalamic-pituitary-adrenal axis, which increases vulnerability to stress and is associated with withdrawal-induced anxiety and dysphoria [55].

We also examined the association of the SURPS scores with hazardous alcohol use as defined using the AUDIT cut-off value of 8. Most prior studies examined the SURPS properties in adolescents and young adults (e.g. university students). In our study participants were older (mean age 37.22 ± 0.78 years), thus we attempted to extend the knowledge of the SURPS properties in adult population. We found that female hazardous users of alcohol scored higher on the SURPS impulsivity and sensation seeking subscales. These findings are congruent with a study from Spain, which also measured hazardous alcohol use by the AUDIT [30]. Other studies using the SURPS scale demonstrated that personality relation with increased risk for alcohol use and abuse were reliant on population and alcohol use measurements [51]. Higher impulsivity and sensation seeking was a common finding in adolescent and young adults with alcohol use and abuse [9, 25, 27, 45, 51, 52]. Impulsive traits and sensation seeking as measured by the SURPS were also linked to higher AUDIT scores [24] and for alcohol use and abuse [27, 51, 52]. Absence of internalising trait association with alcohol abuse was consistent with some studies on adolescents and young adults [24, 27, 30, 51].

Most of prior studies using the SURPS have adjusted their analyses by gender [9, 24, 27, 51, 52], but there is a lack of SURPS studies that provided the gender separate analysis on sensitivity. The divergent findings of personality trait relation to gender alcohol use was demonstrated in France [25]. Few studies demonstrated that males scored more on sensation seeking and females more on anxiety sensitivity or hopelessness [9, 24, 25, 30]. However, in some studies there were no differences in SURPS scores between genders [51].

Different patterns of alcohol use/abuse between genders are well known. Males tend to use alcohol more frequently and more hazardously [34]. One of the systems of alcohol abuse profiling, the Cloninger typology, has shown that personality types of risk of alcohol abuse interact with gender, with harm avoidance being more explicit for females and motives of sensation seeking for males [56]. Therefore, the differences of explication of personality traits as measured by the SURPS and knowledge of divergent pathways in addiction disorders for genders encouraged us to perform gender separate analyses of SURPS external validity. Here, gender separate analysis for alcohol dependence suggested that hopelessness was associated with alcohol dependence in males but not females. Motives of consumption of alcohol as a coping tool have previously been demonstrated to be more frequent for males [34].

In comparison of groups of hazardous alcohol uses and control, gender separate analysis demonstrated that associations of sensation seeking and impulsivity with hazardous alcohol use were driven by females. These findings suggest that the SURPS was more sensitive for females on hazardous alcohol use in our study. However, it is important to notice that Lithuania has overall high alcohol consumption that is driven by males [57]. Therefore, hazardous alcohol use for Lithuanian males could be predisposed more by cultural rather than inherited or personality factors.

### Strengths and limitations

Most of the research on the SURPS scale included adolescents or student samples and only few studies included patients with clinical diagnosis of addiction. We demonstrated an appropriate validity of SURPS scale in clinical sample. Knowledge of the SURPS validity and especially of personality traits expression in alcohol dependence would be beneficial for research studies and subsequent choices for personalized treatment methods [56]. Efficacy of current substance disorders treatment methods is poor, only 30% rate is reached when treatment success is evaluated as staying in abstinence [58], although personalised treatment approaches were demonstrated to be more efficient [5, 59].

Moreover, we provided a gender separate analysis for evaluation of SURPS sensitivity. Our results suggested that SURPS scale was more sensitive for females on discriminating hazardous alcohol use. However, the sample size in our study was limited, thus results for gender differences should be interpreted with caution.

## Conclusions

We demonstrated an appropriate validity of the Lithuanian translation of the SURPS scale. The SURPS demonstrated good discriminating value for alcohol dependence in our sample. Persons with a diagnosis of alcohol dependence scored higher on the SURPS subscales of hopelessness, anxiety sensitivity and impulsivity. Hazardous alcohol use was associated with impulsivity and sensation seeking, but these associations were driven by females.

However, two questions (items 6 and 22) were removed from the scale to achieve appropriate construct validity and there was a configural gender non-invariance of the scale. Therefore, our results should be interpreted with caution and studies re-evaluating factor structure of the SURPS in Lithuanian populations are encouraged. Nevertheless, the SURPS demonstrated appropriate sensitivity for discriminating alcohol dependence, suggesting that it could be useful for selecting personality targeted alcohol misuse prevention and treatment approaches.

## Data Availability

All necessary data is included in the manuscript. Additional data is available from corresponding author upon request**.**

## Abbreviations

SURPS: Substance Use Risk Profile Scale
AUDIT: Alcohol Use Disorder Identification Test
ICD – 10: International Classification of Diseases – 10
DSM-5: Diagnostic and Statistical Manual of Mental Disorders – 5
MMPI: Minnesota Multiphasic Personality Inventory
TPQ: Tridimensional Personality Questionnaire
APP: Addiction Prone Personality Scale
BIS − 11: Barratt Impulsiveness Scale – 11
TIPI: Ten Item Personality Measure
PHQ-4: Patient Health Questionnaire - 4
WHO: World Health Organization
EFA: Exploratory factor analysis
CFA: Confirmatory factor analysis
CFI: Comparative Fit Index
RMSEA: Root Mean Square of Approximation
NFI: Normed Fit Index
ROC: Relative operating characteristic curve
AUC: Area under the curve
SD: Standard deviation
df: Degrees of freedom
Q1: Quartile 1
Q3: Quartile 3
